# Morphometric and total protein responses in *Meloidogyne incognita* second-stage juveniles to Nemafric-BL phytonematicide

**DOI:** 10.1038/s41598-020-80210-7

**Published:** 2021-01-13

**Authors:** Phatu W. Mashela, Ebrahim Shokoohi

**Affiliations:** Green Biotechnologies Research Centre of Excellence, University of Limpopo, Private Bag X1106, Sovenga, 0727 Republic of South Africa

**Keywords:** Zoology, Animal behaviour

## Abstract

After hatch, second-stage juveniles (J2) of root-knot (*Meloidogyne* species) nematodes could spend at least 12 weeks in soil solutions searching for penetration sites of suitable host plants. The external covering of nematodes, the cuticle, consists of various layers that contain glycoproteins, lipids, soluble proteins (collagens) and insoluble proteins (cuticulins). Generally, cucurbitacins are lipophilic, but there is scant information on how cuticular proteins relate to these complex terpenoids. A study was conducted to investigate the nature and extent of damage post-exposure of J2 to a wide range of Nemafric-BL phytonematicide concentrations. Post-72 h exposure to Nemafric-BL phytonematicide, nematode morphometrics versus phytonematicides exhibited either negative quadratic, positive quadratic, or negative linear relations, with the models explained by significant (*P* < 0.05) associations (R-squared). Similarly, total proteins versus phytonematicide exhibited significant negative quadratic relations. The principal component analysis indicated that concentration level of 2–4% of Nemafric-BL phytonematicide have the highest impact on the morphometric changes of J2. In conclusion, the nature and extent of damage suggested that Nemafric-BL phytonematicide was highly nematicidal as opposed to being nematostatic, thereby explaining its potent suppressive effects on nematode population densities.

## Introduction

Post-withdrawal of environment-unfriendly fumigant synthetic nematicides from the agrochemical markets in 2005^1^, crop losses due to plant-parasitic nematodes increased^2,3^, with global estimates rising to as high as 37% at eight-years relative to the pre-withdrawal year of methyl bromide^4^. A wide range of alternatives were then researched and developed for managing nematode population densities^5^, particularly the notorious sedentary root-knot (*Meloidogyne* species) nematodes^4^. Among the alternatives for managing nematode population densities were the cucurbitacin-containing phytonematicides, which consistently reduced high percentages of nematode numbers relative to positive controls^5^. At excessively low concentrations, these phytonematicides were shown to promote juvenile hatch but inhibited the activity at high concentrations^6^. Nemarioc-AL and Nemafric-BL phytonematicides contain cucurbitacin A (C_32_H_46_O_9_) and cucurbitacin B (C_32_H_46_O_8_) active ingredients, respectively^7^. The two phytonematicides are being produced from fruits of wild cucumber (*Cucumis myriocarpus* Naude.) and wild watermelon (*Cucumis africanus* L.), respectively^5^.

The second-stage juveniles (J2) of *Meloidogyne* species versus increasing concentration of the two phytonematicides have been consistent in exhibiting negative quadratic or linear relations. The J2 under various conditions are highly sensitive to the products^5^. Each product affects J2 in soil solutions and roots^8^, with the highest effects occurring in soil^5^. In some cases, after hatching the J2 of *Meloidogyne* species could spend as long as 12 weeks in the active form without a suitable host^9^. Upon following chemical cues from and penetrating the roots^10^, J2 move through the cortex towards the root tips and after reaching the tips, penetrate the vascular bundle and move upward to the infection site^11,12^. During exploratory movements in the soil, J2 take as long as five days to 12 weeks^4,9^, where lipid-using J2 could be exposed to cucurbitacins in soil solutions. Infective juveniles (IJ) of an entomopathogenic nematode, *Steinerma feltiae* Filipjev, were tolerant to cucurbitacin-containing phytonematicides^13,14^, which was confirmed through morphometric measurements^15^. Morphological and cuticular responses of plant-parasitic nematode J2 during exposure to cucurbitacin-containing phytonematicides had not been reported. The objective of this study, therefore, was to investigate the morphometric and total protein responses of *M. incognita* J2 to increasing concentration of Nemafric-BL phytonematicide.

## Results

The Shapiro–Wilk test was performed on standardized residuals to test for deviations from normality^16^, while the Levene test^17^ was used to test for homogeneity of treatment combination variances for sequential experiments. The standardized residuals were acceptably normal with homogeneous treatment variances. Consequently, morphometric (n = 70) and total protein (n = 42) data were pooled and subjected to analysis of variance using SAS software. Treatment effects were highly significant (*P* < 0.01) on all variables except for the significant (*P* < 0.05) effects on lip region width and median bulb width. All data were subjected to lines of the best fit using Microsoft Excel.

### Negative quadratic relations

**Figure 1:**
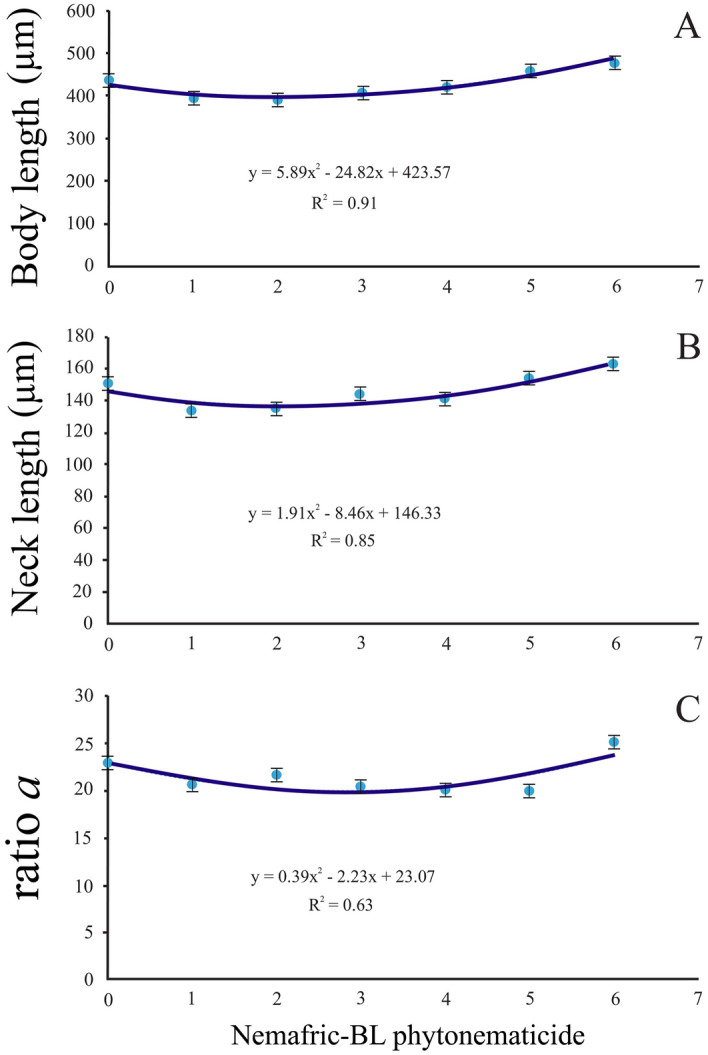
Negative quadratic relations depicting body length (**A**), neck length (**B**) and De Man ratio a (**C**) of *Meloidogyne incognita* second-stage juveniles post-exposure to Nemafric-BL phytonematicide: Measurements show standard deviations.

The body length, neck length, and De Man ratio a versus increasing concentration of Nemafric-BL phytonematicide each exhibited negative quadratic relations (Fig. 1A–C). The models for the three variables were explained by 91, 85 and 63% associations, respectively. Using the x = –b_1_/2b_2_ relation from the Y = b_2_x^2^ + b_1_x + c quadratic equation^5^, the minimum body length, neck length and De Man ratio a accrued at 2.11, 2.21 and 2.82% phytonematicide, respectively.

### Positive quadratic relations

**Figure 2:**
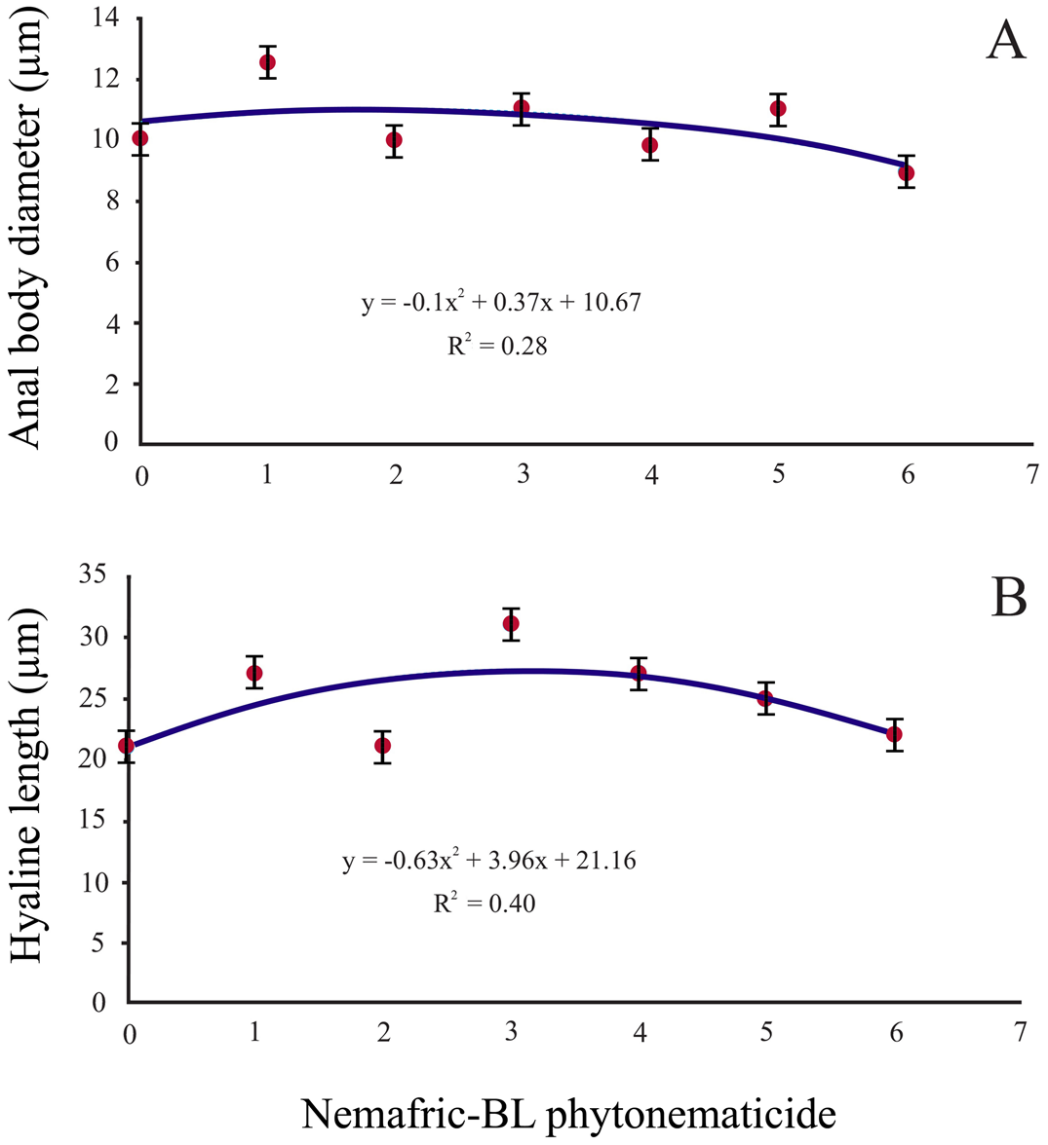
Positive quadratic relations depicting anal body diameter (**A**) and hyaline length (**B**) of *Meloidogyne incognita* second-stage juveniles post-exposure to Nemafric-BL phytonematicide. Measurements show standard deviations.

In contrast, anal body diameter and hyaline length versus the phytonematicide concentration each exhibited positive quadratic relations, with the models explained by 28 and 40% associations, respectively (Fig. 2A,B). The maximum anal body diameter and hyaline length were achieved at 1.86 and 3.14% phytonematicide concentrations, respectively.

### Negative cubic responses

**Figure 3:**
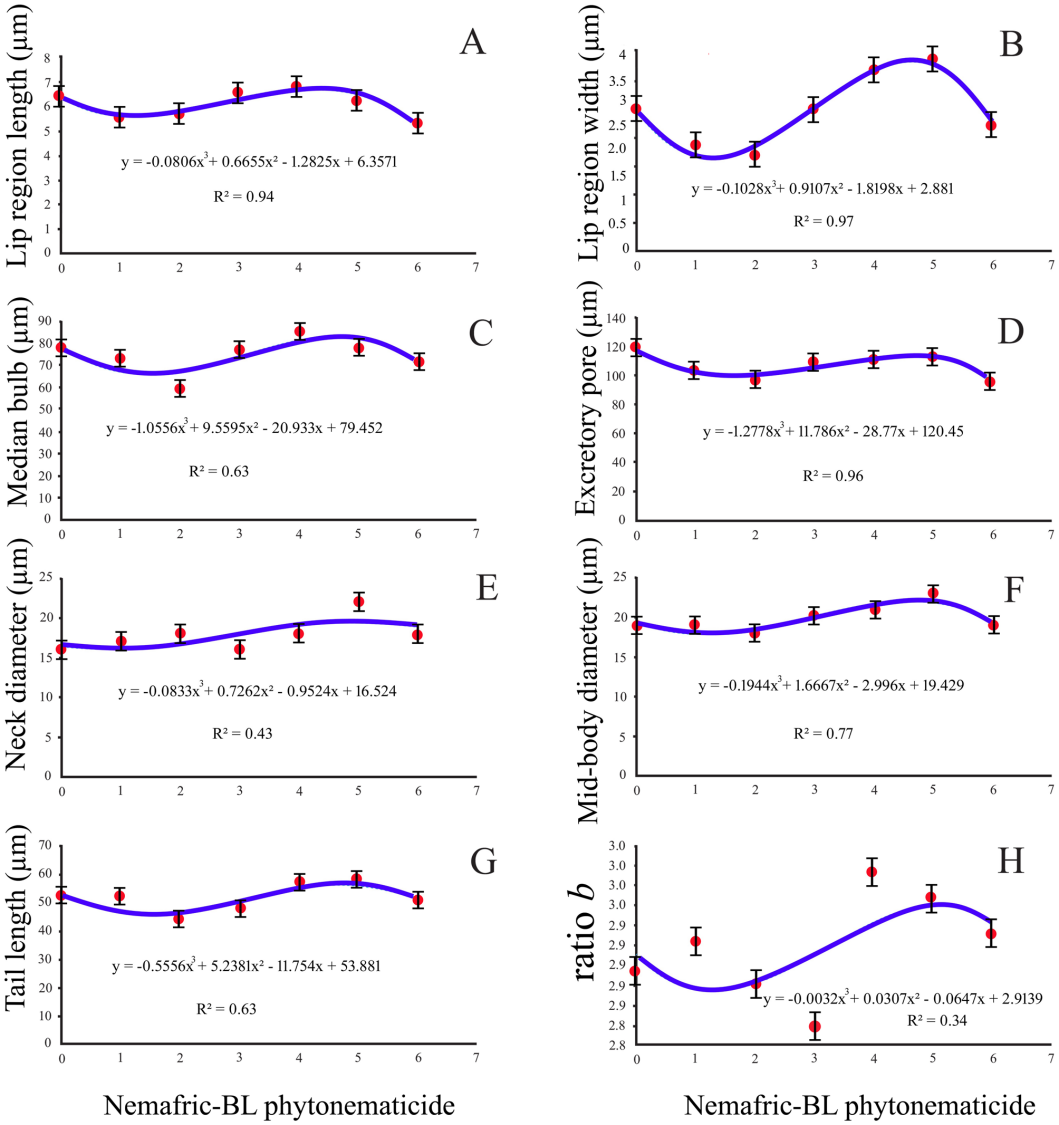
Negative cubic relations depicting lip region length (**A**), lip region width (**B**), anterior end to median bulb (**C**), excretory pore to anterior end (**D**), neck diameter (**E**), mid-body diameter (**F**), tail length (**G**) and De Man ratio b (**H**) of *Meloidogyne incognita* second-stage juveniles post-exposure to Nemafric-BL phytonematicide. Measurements show standard deviations.

Lip region length, excretory pore to anterior end, anterior end to the median bulb, tail length, mid-body diameter, neck diameter, lip region width, and ratio b versus phytonematicide concentration each exhibited negative cubic relationship (Fig. 3A–H). The respective models were explained by 94, 96, 63, 63, 78, 43, 98, 0.35 and 77% associations, respectively.

### Positive cubic relations

**Figure 4:**
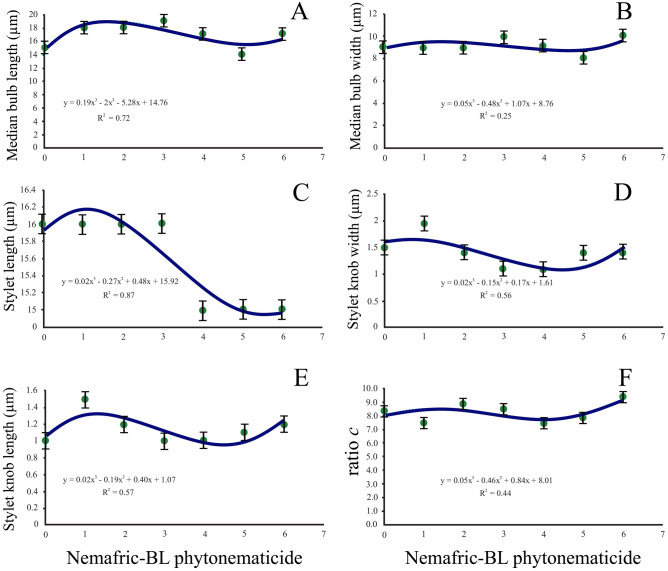
Positive cubic relations of *Meloidogyne incognita* morphometrics versus Nemafric-BL phytonematicide: (**A**) Median bulb length, (**B**) median bulb width, (**C**) stylet length, (**D**) stylet knob width, (**E**) stylet knob length and (**F**) c index. Measurements show standard deviations.

Median bulb length, stylet length, stylet knob length, median bulb width, stylet knob width, and De Man ratio c versus phytonematicide concentration each exhibited positive cubic relations (Fig. 4A–F). The respective models were explained by 73, 88, 58, 25, 56 and 45% associations.

### Graphic morphological changes

**Figure 5:**
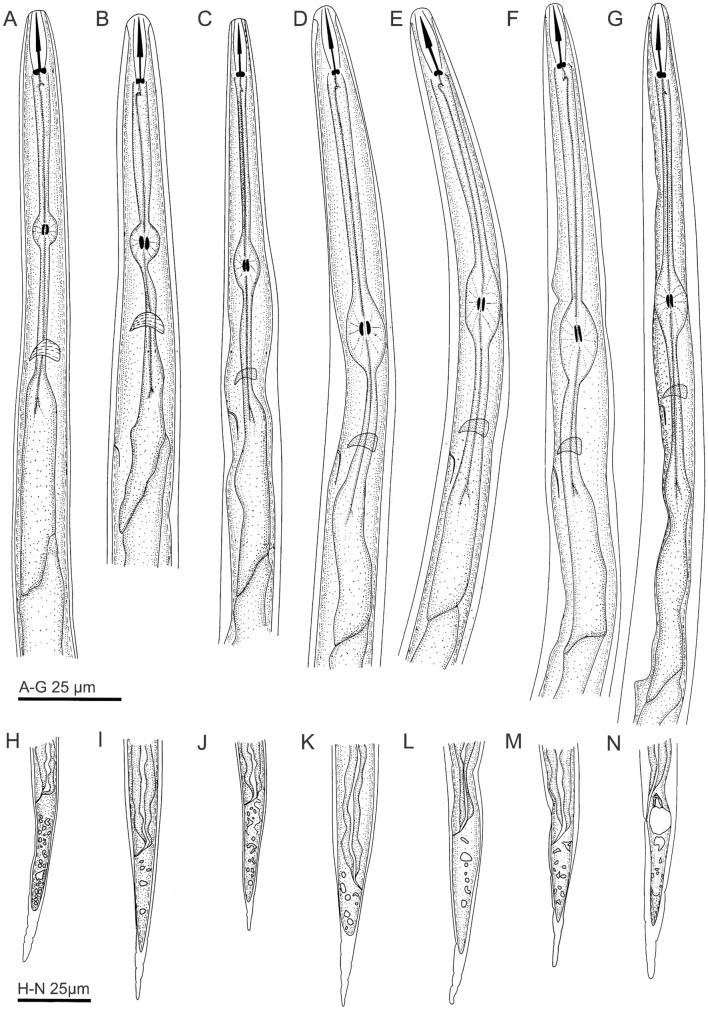
Morphological changes of *Meloidogyne incognita* post-exposure to Nemafric-BL phytonematicide. Anterior end (**A**–**G**), posterior end (**H**–**N**): (**A**), H: 0%; (**B**), I: 2%; (**C**), J: 4%; (**D**), K: 8%; (**E**), L: 16%; (**F**), M: 32%; (**G**), N: 64% Nemafric-BL phytonematicide.

Anteriorly, distinct graphic morphological changes as phytonematicide concentrations increased from 0 to 64% included elongated pharynx length, and disfigured median bulbs (Fig. 5A–G). Posteriorly, there were reduced lipids in tails and reduced hyaline (Fig. 5H–N).

### Total protein analysis

**Figure 6:**
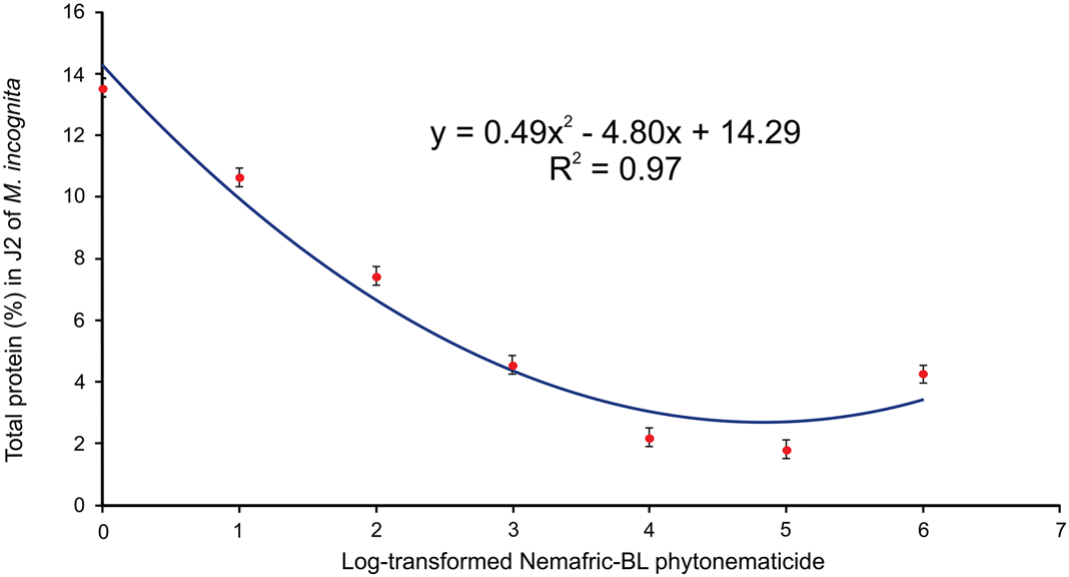
Total protein of *Meloidogyne incognita* post-exposure to Nemafric-BL phytonematicide.

Total protein (%) and Nemafric-BL phytonematicide exhibited negative quadratic relations, with the model explained by 97% association (Fig. 6). As described in morphometric variables, the minimum total protein was reached at 4.82% phytonematicide concentration, a much higher concentration than those of most other variables.

### Principal component analysis

**Figure 7:**
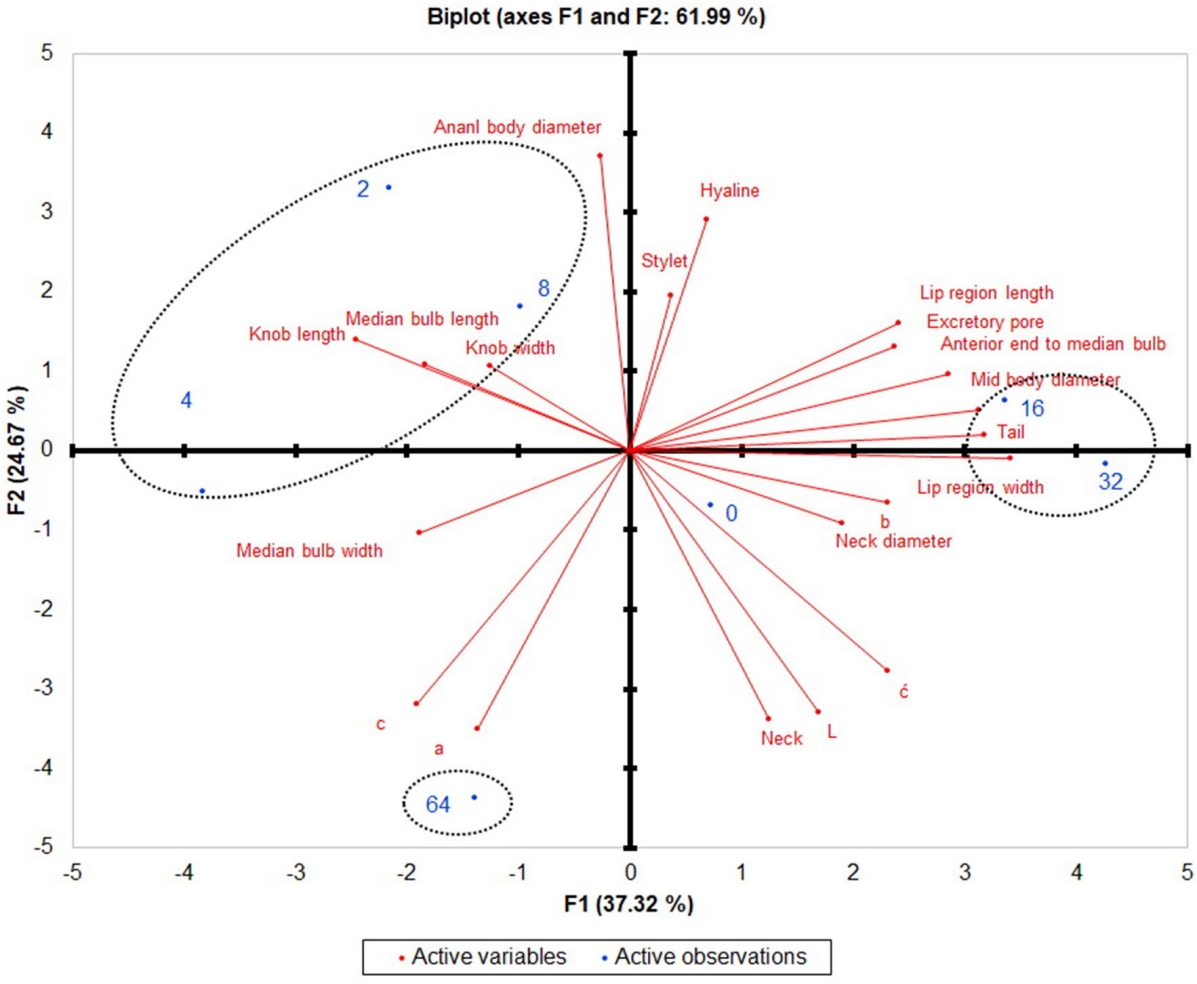
Two-dimensional plot of principal component analysis (PCA) of morphometric changes of *Meloidogyne incognita* exposed to different concentration of Nemafric-BL phytonematicide.

The PCA results showed morphometric variation in the J2 of *M. incognita* exposed to Nemafric-BL phytonematicide. An accumulated variability of 61.99% was observed in the J2 (Fig. 7). Regarding the morphometric changes, lip region width (0.980) and tail length (0.912) showed the highest positive coefficient correlations among the different concentrations of Nemafric-BL phytonematicide and were responsible for the variability of the F1. However, anterior end to median bulb length (− 0.707) and c value (− 0.547) displayed negative coefficient correlation to the phytonematicide F1 (Fig. 7). The highest coefficient correlation with F2 was observed in anal body diameter (0.864) and hyaline portion of the tail (0.677) (Table 1). The result of the factor score indicated that 16% (3.365) and 32% (4.266) of Nematfric-BL phytonematicide had the most positive effect on the morphometric changes (Table 1). The most important character which was being affected in Nemafric-BL phytonematicide is the tail length.

**Table 1:** Principal component analysis of Nemafric-BL phytonematicide concentrations and *M. incognita* variables.

	Factor loadings of the variables		Concentration	Factor score of the concentrations	
	F1	F2		F1	F2
L	0.485	− 0.770	0	0.726	− 0.689
a	− 0.394	− 0.818	2	− 2.155	3.310
b	0.662	− 0.153	4	− 3.827	− 0.507
c	− 0.547	− 0.746	8	− 0.982	1.810
ć	0.662	− 0.650	16	3.365	0.625
Lip region width	0.980	− 0.021	32	4.266	− 0.172
Lip region length	0.691	0.374	64	− 1.394	− 4.377
Median bulb width	− 0.544	− 0.241			
Median bulb length	− 0.707	0.326			
Stylet	0.106	0.454			
Knob width	− 0.360	0.248			
Knob length	− 0.528	0.253			
Excretory pore	0.681	0.306			
Neck	0.357	− 0.789			
Anterior end to median bulb	0.819	0.225			
Anal body diameter	− 0.077	0.864			
Mid body diameter	0.898	0.119			
Neck diameter	0.548	-0.213			
Tail	0.912	0.047			
Hyaline	0.197	0.677			

## Discussion

The negative quadratic responses had minima morphometric effects on body length, neck length, and ratio a (body length/mid-body diameter) from 2 to 3% Nemafric-BL phytonematicide. This range agrees with that empirically-established for managing nematode population densities of *Meloidogyne* species on various crops^18,19^. The optimum minimum for a morphometric effect depicts the phytonematicide concentration where the physiological functionalities of the test organ cease, depicting complete paralysis as observed in fluopyram synthetic chemical nematicide^20^. In contrast, the body length of *S. feltiae* responded to increasing phytonematicide concentration through stimulation, neutral, and inhibition phases, from 0 to 64% Nemafric-BL phytonematicide, the process referred to as density-dependent growth (DDG) patterns^5,21^. In such cases, the optimum maximum depicts the advent of paralysis. In contrast to the negative DDG patterns for body length in *Meloidogyne* species, in *S. feltiae* IJ, the DDG patterns for body length were positive^15^, which is a common feature in phytochemicals, referred to as allelochemicals^21^.

In DDG-associated relations, it is essential to note that the direction of DDG patterns is concentration-specific for the test entity. For instance, if a concentration range is restricted to the stimulation phase for the test entity, the response would be positive linear relation. In contrast, within the inhibition phase, the response would be a negative linear relation^5^. In the range of the neutral phase, there would be no relationship between the dependent and independent variables. For the DDG patterns to occur, the entity should be exposed to concentration ranges that straddle all three phases^5^. Depending on the sensitivity of the test entity to phytonematicides, the response could start at any of the three phases. In the current study, the body length, neck length and De Man ratio a versus phytonematicide, each started at the inhibition phase, suggesting that the variables were highly sensitive to the test product. During exposure of *S. feltiae* to Nemafric-BL phytonematicide from 0 to 64%, the body length-phytonematicide relation started at the stimulation phase. The latter suggests that the IJ were tolerant to the phytonematicide, which can be attributed to their intrinsic survival abilities. Generally, *S. feltiae* IJ are more tolerant to adverse conditions than their parasitic counterparts inside hosts. In contrast, after hatch, J2 of *Meloidogyne* species do not have such survival proficiencies, and therefore, exposure results in immediate inhibition of population densities.

In *S. feltiae* IJ, at low phytonematicide concentration, the body length was elongated due to the simultaneous contraction of somatic longitudinal muscles on the latero-ventral and latero-dorsal sides of the nematode body^22^. However, in *M. incognita* J2, without survival capabilities, the simultaneous relaxation of somatic longitudinal muscles on both sides occurred in response to complete paralysis, characterized by irreversible immobility. In an electron microscopy study^23^, it was shown that a 10-day exposure of *M. incognita* J2 to active ingredients of cucurbitacin-containing phytonematicides at concentrations used in the management of nematodes, cucurbitacin A and B “peeled off” the cuticle from the hypodermal layer, resulting in wrinkled and shrunken bodies^23^. As confirmed by the degradation of total proteins in the current study and the “peeling off”^23^, the nematode cuticle is the first line of defense against cucurbitacins in soil solutions. The cuticle comprises a set of five layers^24^, with the outermost, the epicuticle, consisting of lipids that are coated with glycoproteins^24^. After the epicuticle, the next three layers, namely, the cortical, medial and basal layers, are formed by collagen, which constitutes more than 80% total proteins of the cuticle that are readily soluble in reducing agents^24^. The cortical layer also contains cuticulins, which are insoluble proteins. Currently, it is not known whether the cucurbitacins also reduce the cuticulins at high concentrations, where total proteins start to increase. Generally, the cucurbitacins are lipophilic^25^ and therefore, the epicuticle with its lipids^24^ predisposes the cuticle to attack by the cucurbitacins. The structure of the cuticle, with its collagen struts serving as pillars that separate the cortical and the basal layers, with fluid-filled spaces^24^, confer the nematode cuticle some resilience to stretch longitudinally by modifying angles of the fibers in the outer section of the basal layer^22,24^. The latter could, however, not explain the observed elongated bodies in *M. incognita* when exposed to high concentrations of Nemafric-BL phytonematicide. Since the cuticle confers a hydrostatic skeleton to the nematode body^26^, “peeling off” of the cuticle with the subsequent influx of water could, to a certain degree, explain the observed changes in the body length of *M. incognita* J2.

The positive quadratic responses for anal body diameter as phytonematicide concentrations increased were congruent with decreases in nematode body length. As a general principle, as J2 body lengths decrease at low phytonematicide concentrations, diameters of openings such as anal openings would increase. In contrast, at a high concentration as body length increased, the diameter of openings decreased. The observation agreed with those in *S. feltiae* IJ-phytonematicide relations^15^. Such dynamics, as emphasized in the current study, are intended to regulate the potentially destructive effects of the hydrostatic pressure in the pseudocoelom of the nematode bodies^15^. The decrease in hyaline length of *M. incognita* corresponded with the reduction in the body length, whereas an increase in body length as concentration increased corresponded with an increase in hyaline length. Changes in hyaline length are proportional to those of tail length. In *S. feltiae* IJ, it was shown that changes in body length were inversely proportional to changes in tail length as an adaptation to retain the hydrostatic pressure constant^15^.

Cubic responses in morphometrics of *Meloidogyne* J2 versus phytonematicide concentration exhibited either negative (lip region length, excretory pore to anterior end, anterior end to median bulb, tail length, mid-body diameter, neck diameter, lip region width, De Man b and c' ratios versus phytonematicide concentration) or positive (median bulb length, stylet length, stylet knob length, median bulb width, stylet knob width and De Man c ratio versus phytonematicide concentration) relations. In both cases, relatively high R^2^ values were observed, suggesting the existence of intricate mechanisms that regulate hydrostatic pressure in the pseudocoeloms. It should be noted that the extent of responses in any single variable to the test phytonematicide was concentration-specific. Generally, at low concentration responses were gradual, whereas as concentrations increased, they became instantaneous, possibly to avoid the “shock” effects. The nematode body is “structurally wired” with the nervous system, which includes a range of sensory organs over the entire body, with the highest distribution being anteriorly located^22^. The J2 of plant-parasitic nematodes cannot adjust hydrostatic pressure in their pseudocoeloms for survival when exposed to the low concentration of test phytonematicides used in nematode management. Possibly, J2 have the ability to respond instantaneously to cucurbitacins due to their degradative nature on the cuticle, thereby avoiding tolerance mechanisms that could prolong the pain in J2.

In the early stages of nematode development, particularly during the egg-stage, plant-parasitic nematodes have various survival strategies induced by chemical cues or lack thereof from roots^27^. For instance, the first-stage juveniles (J1) of *Meloidogyne* species inside eggs have innate abilities to enter the dauer phase^28^, which accord J2 hatch to occur as a sequential process instead of being an instantaneous event. The former avoids simultaneous exposure of J2 to unfavorable environmental conditions such as active ingredients of cucurbitacin-containing phytonematicides. Once J2 start to hatch under appropriate chemical cues from root exudates, J2 move through soil solutions searching for suitable penetration sites at the root zones of elongation^10,26^. After penetration, J2 move towards the apex of the root and then turn upward through the vascular bundle to the feeding sites^10^, where the latter are established through the formation of giant cells^10^. As feeding activities are assumed, development from J2 through successive molting to J4 leads to young adults that complete morphological development, culminating in reproduction^12^. Active ingredients of the test phytonematicides have been shown to suppress J2 and eggs inside roots^5^, proving the potent nematicidal attributes of the products in vitro and in vivo.

In other highly specialized plant-parasitic nematodes such as *Anguina tritici* (Steinbuch, 1799) Chitwood, 1935, *Ditylenchus dipsaci* Kuhn, 1857, *Globodera rostochiensis* (Wollenweber, 1923) Behrens, 1975, *Heterodera schachtii* Luc & Memi, 1963, etc., after hatch, J2 could enter hydrobiosis. The latter is a survival form of cryptobiosis, which is induced by gradual dehydration^28,29^. Several other forms of cryptobiosis, namely, chemiobiosis, osmobiosis, anoxybiosis, and thermobiosis, have been widely investigated^28^. Most importantly, when in cryptobiosis, nematodes are tolerant to most unfavorable environmental conditions^28^, presumably, inclusive of the test phytonematicides, but which, due to their potent nature, warrant further investigation. Although studies consistently showed that the test phytonematicides have bioactivities that suppress population densities of *Meloidogyne* species^5,8,18,19,29^ and the citrus nematode, *Tylenchulus semipenetrans* Cobb 1913^30^, the concurrent structural damage and protein degradation as the mode of action are being explained in our study as the first record. This, therefore, opens a new research niche for cucurbitacin-gene interactions in the use of cucurbitacin-containing phytonematicides in the management of plant-parasitic nematodes.

The observed changes, either quadratic or cubic, at various concentrations of Nemafric-BL phytonematicide, constituted notable morphological changes in J2 of *Meloidogyne* species. Results of the study demonstrated that *Meloidogyne* species did not have survival capabilities after J2 hatch, with sensitivities manifested through the structural body and chemical changes that were concentration-specific. The damage to various morphological structures and total proteins suggested that the cucurbitacin-containing phytonematicides could be highly lethal to J2 and, therefore, warranting classification as nematicides as opposed to being nemastotic products. The environment-friendly phytonematicide can have effects on the gene expression^31^, but without affecting the DNA sequencing. Observations in the current study open a new avenue for investigating nematode-cucurbitacin interactions at a gene level.

## Materials and methods

### Preparation of the phytonematicide

Mature fruit at 92 days after transplanting seedlings were harvested from a cultivated field of *C. africanus*, washed using chlorine-free tapwater, cut into pieces and dried at 52 °C for 72 h^5^. Dried fruit were ground in a Wiley mill to pass through a 1-mm-pore sieve. Approximately 40 g *C. africanus* ground materials were fermented in a hermetically-sealed 20-L-plastic container using effective microorganisms (EM) at 30 °C for 14 days until pH was at 3.7^5^. The EM consisted of yeast, photosynthetic bacteria, lactic acid bacteria, actinomycetes, and fermenting fungi^32^. Post-fermentation, a 1000 ml sample was passed through a Whatman 1442-125 Ashless Grade 42 Quantitative Filter Paper.

### Preparation of second-stage juveniles

A population of *M. incognita* was cultured on a 3-week-old susceptible tomato (*Solanum lycopersicum* L.) cv. ‘Floradade’ in ten 20-cm-diameter plastic pots from single egg mass collected from infected greenhouse-raised kenaf (*Hibiscus cannabinus* L.) plants. Sixty days after inoculation, tomato roots were rinsed in 1% NaOCl solution and egg masses were dislodged using a toothpick into a 25-ml glass beaker containing 20 ml distilled water. Eggs and J2 were incubated on filter papers in seven Petri dishes each with ca. 150,000 eggs and J2 per 5 ml water in the growth chamber at 25 ± 2 °C for 96 h to allow for J2 hatch^33^. Phytonematicide stock solutions were diluted in distilled water and pipetted into a 96-well plate to 0, 4 8, 16, 32, and 64 µg ml^−1^ phytonematicide, each concentration level admixed with ca. 1050 J2 in each well.

### Morphometric and drawings of the second-stage juveniles

Post-exposure, each treatment was diluted ten times using distilled water and a 50 µl solution with J2 isolates per treatment was placed in wells of a clean 96-well plate. Specimens were fixed on mounting slides^34^. Morphometric data were collected using an Omax light microscope equipped with a measuring software program. Variables measured included length (body, lip region, median bulb, stylet, knob, excretory pore to anterior end, neck, anterior end to median bulb, tail, hyaline), diameter (anal body, mid-body, neck), width (lip region, median bulb, knob) and De Man indices a, b and c. Drawings were made according to the LM pictures captured using a camera attached to an Omax microscope. Two sequential experiments for collecting morphometric data were conducted bi-monthly.

### Determination of total proteins

*Meloidogyne incognita* J2 were prepared and exposed to seven concentrations of Nemafric-BL phytonematicides as described in morphometric experiments, and each treatment replicated three times. After dilution in distilled water, approximately 1000 J2 (~ 0.15–0.20 g) in distilled water were weighed into a tin foil cup and then placed into an automated sample loader of TruSpec CHNS Macro (Leco, St. Joseph, MI, USA) instrument. The loader dropped the sample into a hot furnace (950 °C), which was flushed with oxygen for rapid and complete combustion. The products of combustion were passed through a secondary furnace, termed afterburner (850 °C), for further oxidation and removal of particles. After that, the materials were collected in a collection vessel, with a representative aliquot transferred to the helium carrier flow, which was then swept through a hot copper to convert the nitrogen oxides into nitrogen and then through Lecosorb (sodium hydroxide) and Anhydrone (magnesium perchlorate) to remove carbon dioxide and water, respectively. The nitrogen was then quantified by passing the gas through a thermal conductivity cell, which emitted electrical signals proportional to the nitrogen content. The entire process per sample was approximately 4 min. The final results were calculated from a calibration curve plotted using ethylenediaminetetraacetic acid (EDTA) as the nitrogen calibration standard. Two sequential experiments were conducted monthly.

### Data analysis

Data were subjected to analysis of variance (ANOVA) using SAS software^35^. Data with significant (*P* < 0.05) treatments were further subjected to lines of the best fit. Prior to lines of the best fit, treatments (0, 2, 4, 8, 16, 32 and 64% dilutions) were expressed as exponentials (2^0^, 2^1^, 2^2^, 2^3^, 2^4^, 2^5^ and 2^6^) and transformed using log_2_2^x^ = x (log_2_2) = x(1) = x to homogeneous the variances^36^. Morphometric data and total protein (S1 and S2, supplementary tables, respectively) versus phytonematicide concentration were each subjected to lines of the best fit using Microsoft Excel 2016. Findings were grouped and discussed based on dose–response growth patterns, with the generated curves being improved using the Photoshop M.E. program. Principal component analysis using the Pearson method was done by XLSTAT^37^. Twenty morphometric traits obtained from fixed nematodes including “de Man's indices” (*a*, *b*, *c*, *c'*), body length, lip region width, lip region height, stylet length, knob width, knob length, median bulb width, median bulb length, anterior end to middle of median bulb, neck length, excretory pore to anterior end, neck base diameter, mid body diameter, anal body diameter, tail length and hyaline portion of the second stage juvenile were used for PCA analysis of *M. incognita*. The measures were normalized through XLSTAT software prior to their analysing^37^. The scores values were determined for each isolate based on each of the principal components, and the scores for the first two components were used to form a two-dimensional plot (F1 and F2) of each concentration of Nemafric-BL phytonematicides based on eigenvalue given by the software XLSTAT.

## Supplementary Information

Supplementary Table S1.

Supplementary Table S2.
